# The Association Between Alexithymia, Interoception, and Pain in Pediatric Chronic Pain: A Systematic Review

**DOI:** 10.1177/27683222251375956

**Published:** 2025-09-24

**Authors:** Alisha Bruton, Hayleigh Ast, Jacqueline R. O’Brien, Heather Zwickey, Anna C. Wilson, Cynthia Price, Dana Dharmakaya Colgan

**Affiliations:** 1 Center for Mental Health Innovation, Department of Psychiatry, Oregon Health & Science University, Portland, Oregon, USA.; 2 Department of Pediatrics, Oregon Health & Science University, Portland, Oregon, USA.; 3 Helfgott Research Institute, National University of Natural Medicine, Portland, Oregon, USA.; 4 Department of Biobehavioral Nursing and Health Informatics, University of Washington, Seattle, Washington, USA.; 5 Department of Neurology, Oregon Health & Science University, Portland, Oregon, USA.

**Keywords:** pain, child, adolescent, interoception, alexithymia, emotion

## Abstract

**Objectives:**

Pediatric chronic pain negatively affects emotional, psychological, and social function. This may be due to differences in interoception, the ability to notice and contextualize sensations arising inside the body, and alexithymia, the inability to identify and communicate emotions, as has been shown in adults. In children with chronic pain, interoceptive impairment include difficulties perceiving, interpreting, or responding to physical symptoms such as hunger or pain. Alexithymia symptoms include limited emotional vocabulary or reliance on complaining about physical symptoms to communicate emotional distress. Both alexithymia and interoceptive impairment could contribute to the development or maintenance of chronic pain and lead to issues with pain management. However, whether these are altered in pediatric chronic pain has not been investigated.

**Methods:**

The protocol was written prospectively and indexed online in the International Prospective Register of Systematic Reviews (PROSPERO; #42023439236). The literature search included the Cumulative Index to Nursing and Allied Health (CINAHL), PsycINFO, PubMed/MEDLINE, Scopus, and Google Scholar data-bases. Eligible studies included participants aged ≤18 years old with chronic pain conditions, assessed for symptoms of alexithymia or interoception. Study screening and data extraction were completed independently in duplicate. PRISMA guidelines were followed; the Newcastle-Ottawa Scale assessed risk of bias.

**Results:**

Fourteen studies assessing alexithymia in children/adolescents with/without chronic pain were identified. Of eight studies that reported mean alexithymia symptoms between groups, six reported significantly higher symptoms in the pain group. Alexithymia symptoms were associated with “pain bother” and “interference” but not “intensity.” Two studies also assessed interoception; one compared pain/non-pain groups, finding a significant association between decreased self-reported interoception and higher chronic pain.

**Conclusions:**

Alexithymia symptoms may be increased in children/adolescents with chronic pain. Our review was limited by incomplete reporting and inconsistent measures across studies, decreasing certainty in the overall strength of the evidence. There is a need to understand the role of interoception and alexithymia in pediatric pain to better mitigate and treat pediatric pain. More research is warranted.

## Introduction

Chronic pain is pain that lasts or recurs for 3 months or more.^1^ Pediatric chronic pain is a significant public health problem that negatively impacts a youth’s physical, psychological, social, and academic functioning^2^ and costs an estimated $19.5 billion annually in the United States.^3^ Understanding underlying factors associated with the experience of pediatric chronic pain is critical to developing effective intervention and prevention programs. Alexithymia and interoception are two related factors thought to be associated with the development and maintenance of chronic pain within adult populations^4-7^; however, they have received little attention in the field of pediatric chronic pain.

Alexithymia is a multifaceted construct consisting of (1) difficulty identifying and describing one’s emotions, (2) difficulty distinguishing emotional feelings from bodily sensations, (3) limited self-reflective thought toward inner experience, and (4) limited imagination and fantasy life.^8^ Three recent systematic reviews demonstrated that adults with chronic pain report higher symptoms of alexithymia when compared with individuals without pain.^9-11^ Previous research has also found symptoms of alexithymia to be associated with greater pain intensity,^9,11,12^ pain interference,^7,9^ and anxiety and depression levels,^9,11^ as well as increased risk for the onset or persistence of chronic pain in adults.^12-14^

Interoception is a related, yet distinct, factor that is understood as the conscious and subconscious processing by which the nervous system senses, interprets, and integrates signals originating from one’s own body,^15^ and it is thought to be critical for maintaining homeostasis.^16,17^ A three-part framework has been proposed: interoceptive accuracy (objective ability to detect bodily signals), interoceptive sensibility (subjective awareness of these signals), and interoceptive awareness (metacognitive insight into accuracy).^18^ Accuracy is measured by objective methods such as heartbeat counting, sensibility by self-report questionnaires, and awareness by how confident a person is in their judgment of performance in measures of interoception and how closely this judgment aligns with their actual performance.

Two systematic reviews and one meta-analyses have examined the role of interoception in adults with chronic pain, all of which reported reduced interoceptive accuracy in adults with chronic pain, compared with adults without.^4-6^ One of the reviews also concluded that within chronic pain conditions such as musculoskeletal and neuropathic pain, higher interoceptive sensibility was associated with lower pain severity and frequency in some, but not all, studies.^5^

Importantly, there is a growing theoretical consensus that impairments in processes of interoception play a critical role in the emergence of alexithymia. One theory about the mechanism which connects the two states is that alexithymia may arise from a failure of interoceptive processes.^19^ A meta-analysis of 66 studies examined the association between alexithymia and interoceptive sensibility, awareness, and accuracy.^20^ They reported that alexithymia was moderately negatively correlated with self-reported interoceptive accuracy but not objective interoceptive accuracy (via heartbeat tasks) or interoceptive awareness. Alexithymia was also correlated with interoceptive sensibility, but the strength and direction of the association depended on the questionnaire used.

Thus, research suggests that alexithymia may be associated with difficulties linking physiological indicators of affective arousal with feeling states (e.g., recognizing heart-racing or bodily tension with fear or anxiety) or difficulties discriminating different patterns of affective arousal, which may result in a general awareness of being “upset” but not being able to specify which emotion is being experienced. Within adult populations, interoception is thought to represent a shared transdiagnostic vulnerability that underlies atypical emotional processing in a variety of disparate clinical populations, including those with chronic pain.^15^

Although there is growing evidence that alexithymia and interoception are important factors related to the development and maintenance of chronic pain within adult populations, these factors have received little attention in the field of pediatric chronic pain. Measures of alexithymia^21,22^ and interoception^23^ have been developed and validated in youth; however, to the best of our knowledge, no review exists on alexithymia or interoception in children/adolescents with chronic pain. Understanding how alexithymia and interoception are altered in chronic pain is the critical first step to developing potential therapies to address or prevent both chronic pain symptoms and disorders such as depression and anxiety that occur with, or due to, the chronic pain.^24,25^ A recent theoretical review discussed possible mechanisms by which interoception could be altered in pediatric chronic pain, calling out the importance of examining this connection.^24^ Further-more, alexithymia, interoception, and emotion regulation change with development; thus, results from adult studies may not be applicable to children and adolescents.^26-28^

Therefore, the primary aims of this systematic review include investigating whether: (1) alexithymia and interoception are altered in children/adolescents with chronic pain compared with those without pain and (2) if there is an association between pain and alexithymia or between pain and interoceptive ability (accuracy, sensibility, or awareness) among children/adolescents with chronic pain. Our hypotheses were that alexithymia would be higher and interoception would be lower in children/adolescents with chronic pain compared with those without pain and that measures of pain (severity, disability, etc.) would be negatively associated with interoceptive ability and positively associated with alexithymia.

## Methods

This review was conducted in accordance with the Preferred Reporting Items for Systematic Reviews and Meta-Analyses (PRISMA) checklist,^29^ and a protocol was written prospectively and indexed on the PROSPERO database before the review began (CRD #42023439236). Protocol deviations were recorded (Supplementary Table SA1).

### Search strategy

The literature search included the CINAHL, PsycINFO, PubMed/MEDLINE, and Scopus databases. The search strategies included terms related to alexithymia, interoception, pain, children, and adolescents (Supplementary Appendix SA2). ClinicalTrials.gov, Open Science Framework, and medRxiv were searched for study protocols, from studies in progress or recently completed. PROSPERO was searched for relevant systematic reviews. Google Scholar was searched for relevant citations and articles, including gray literature. As experts in the field, the senior coauthors consulted on search strategy development. The reference lists of all identified studies were hand-searched for other relevant articles. The literature search itself did not have any restrictions on publication date or language. When studies were identified in languages other than English, they were translated using freely available software (Google Translate) when possible. Studies published through July 31, 2025, were included.

### Study selection

#### Study population.

Studies had to include human participants ≤18 years old. If studies included participants both younger than and older than 18 years, they were considered if data for participants ≤18 years old were reported separately from those >18 years old. Studies must have enrolled participants with a chronic pain condition, including, but not limited to, musculoskeletal pain, functional abdominal pain, nerve pain, jaw pain, headache, or migraine. No restrictions were put on duration of pain (i.e., >3 months, >6 months), but these data were extracted for reporting. No other restrictions were placed on participants.

#### Study type.

All study designs were eligible for inclusion in this review, such as clinical trials, case–control studies, cohort studies, observational studies, case reports/series, and open-label studies. Gray literature (unpublished theses, dissertations, etc.) was eligible for inclusion if it otherwise met criteria.

#### Variables of interest.

Studies must have included at least one of the following: self- or proxy-report (parent, teacher, therapist) questionnaires on alexithymia or self- or proxy-report questionnaires or behavioral measures of interoception. Noninterventional studies and studies with any type of intervention were eligible, and studies were not required to have a comparison group, but when they did, data were extracted for both pain and comparison groups. When studies enrolled additional comparison groups (i.e., a group with internalizing disorders), data for those groups were extracted as well.

#### Study eligibility.

If the literature searches identified no or minimal eligible studies for alexithymia, interoception, or both, the review still proceeded as planned, in alignment with recommendations regarding the publication of so-called “empty reviews.”^30-32^ Reviews that identify no eligible studies may still play an important role in clinical research by identifying gaps in the literature to guide funding allocation or justify future trials and by informing clinicians about a lack of evidence about a particular association or treatment effect.^30,31^

#### Study inclusion and data collection

The Rayyan software was used to screen all records identified through the literature searches.^33^ After deduplication, title and abstract screening was done by two or more authors, independently in duplicate or triplicate (A.B., D.C., and H.A.). Full texts were retrieved for any potentially eligible studies and evaluated by two or more independent reviewers (A.B., D.C., and H.A.). Disagreements were resolved by discussion or by consulting an additional author (A.W.). Reasons for excluding each study were noted to be reported in a PRISMA flowchart.

Data extracted from the studies included information on the demographic characteristics of participants, the outcome measures used, study results, and other information (Supplementary Appendix SA1). A data extraction form was made in Microsoft Excel, and data were extracted independently in duplicate by two authors (A.B., H.A.). If any studies require clarification or additional information, authors were contacted via email up to three times.

### Measures.

#### Pain

Studies were not required to have measures of pain characteristics, but these data were included if available. Eligible measures were self- or proxy-report questionnaires on pain characteristics such as pain intensity, pain interference, pain-related disability, and activity limitations. Measures that assessed pain-related cognitions such as pain catastrophizing, pain acceptance, or fear of pain were also eligible for inclusion.

#### Alexithymia

Eligible alexithymia measures included self-or proxy-report measures, including but not limited to the Toronto Alexithymia Scale, the Perth Alexithymia Questionnaire, the Alexithymia Questionnaire for Children (AQC), or adaptations/translations of these.

#### Interoception

Three dimensions of interoception outlined by Garfinkel et al. (2019) were considered for this review: interoceptive accuracy (measured via objective tests such as the heartrate tracking test), interoceptive sensibility (measured via self-report questionnaire such as the Multidimensional Assessment of Interoceptive A wareness, MAIA), and interoceptive awareness (the association between accuracy and sensibility).^18^ Interoceptive measures that targeted any of these three dimensions that were assessed via child self-report, proxy-report, or via behavioral task were included.

#### Quantitative synthesis

Due to the anticipated differences in populations, study designs, measures, and outcomes, a meta-analysis was not planned.

#### Risk of bias

Risk of bias was assessed with the Newcastle–Ottawa Scale for nonrandomized studies,^34^ adapted for crosssectional, case–control studies.^35^ A six-point evaluation rubric was developed by four authors (A.B., H.A., D.C., and A.W.) and used independently in duplicate by two authors (A.B., H.A.); disagreements were resolved by consulting a third and fourth author (D.C., A.W.; Supplementary Table SA2). Cohort studies or those without a comparison group were not evaluated with this tool.

#### Missing data

If a study was eligible for inclusion but had missing data or unclear reporting, up to three attempts were made to contact study authors for clarification.

## Results

### Study selection

**FIG. 1.**
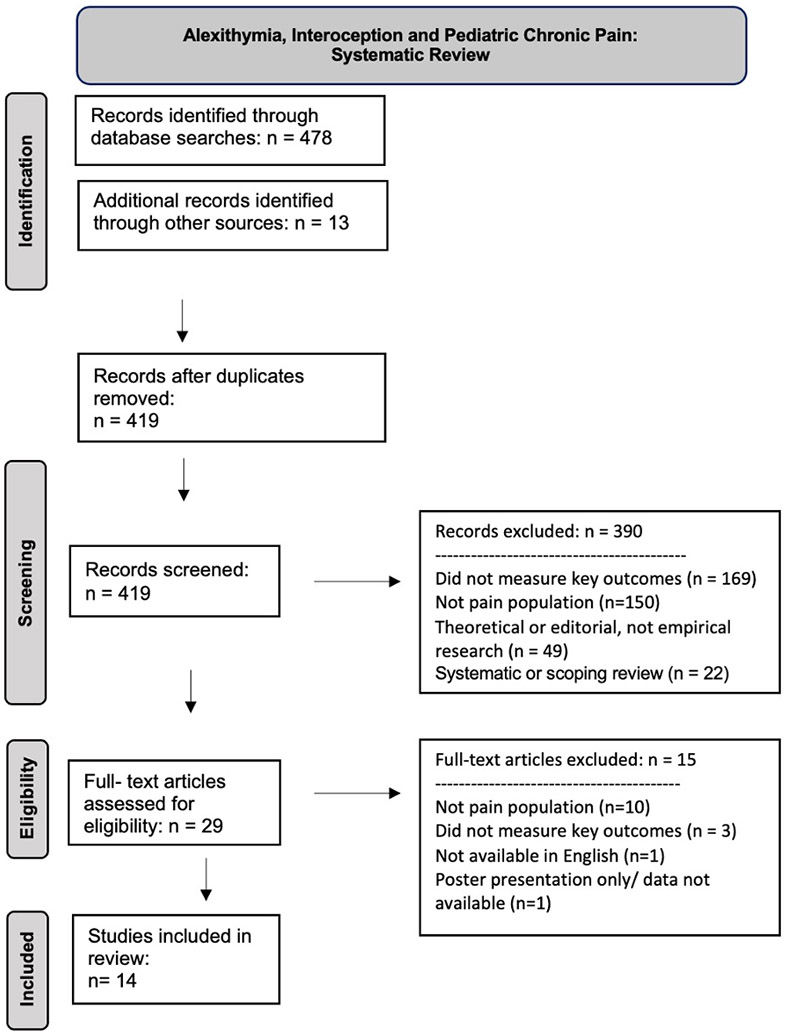
PRISMA diagram.

The systematic literature searches resulted in 686 records (Fig. 1). After removing duplicates, 597 remained. A total of 561 records were excluded after title and abstract screening, and 36 full texts were assessed for eligibility (details in Supplementary Table SA3). Of these, 14 studies on alexithymia and chronic pain in children and adolescents met inclusion criteria, and two of these also had interoceptive outcomes.^36,37^ A systematic review on alexithymia in chronic pain was identified; it included studies in both adults (k = 72) and children (k = 5); all the studies in children have been included in this review.^38^ Six studies on interoceptive exposure interventions for chronic pain in children and adolescents were excluded for not using measures of interoception but are briefly discussed below.

#### Missing data.

Nineteen authors were contacted via email for clarification and/or to request missing data. Of the 14 authors contacted for clarification, nine responded. Missing data were requested for five studies, but only one author responded. One of the studies was originally in French, and although a research librarian was unable to identify an English version, she used the Translate feature in Google Chrome to translate the text, which was then included in this review.^37^

### Alexithymia

#### Study characteristics.

The 14 included studies enrolled participants with chronic pain,^39-41^ persistent somatoform pain disorder,^42^ somatoform symptom disorder,^37^ functional abdominal pain,^36^ fibromyalgia,^43,44^ or headache, including tension-type headache and migraine (Table 1).^45-50^ All studies were crosssectional, survey-based designs, except for one that was a descriptive study of the biopsychosocial characteristics of a sample of adolescents with chronic pain that involved an evaluation by a clinician but no self-report questionnaires.^41^ Most studies enrolled both a pain group and a comparison group without pain, except for studies that enrolled (1) only a pain group,^37,41,50^ (2) a pain group and a fibromyalgia group,^44^ or (3) a pain group and an internalizing disorders group (anxiety or depression).^49^

Nine studies enrolled pain groups that had ≥60% of participants who identified as female. In the other five studies, participants ranged from 41% to 57% female. Most studies used diagnostic criteria to evaluate the pain condition (e.g., the International Classification of Headache Disorders for studies that enrolled participants with headaches), and most also had inclusion criteria for duration of pain (most often 3 or 6 months; Table 1).

#### Measures.

Alexithymia was measured using several self-report questionnaires. The Toronto Alexithymia Scale, 20-item version (TAS-20), was the most commonly used alexithymia questionnaire.^51^ The TAS-20 has 20 items and is reported as a total score and has three subscales: *Difficulty Identifying Feelings, Difficulty Describing Feelings,* and *Externally Oriented Thinking*. Total scores range from 20 to 100, and higher scores indicate more symptoms of alexithymia. Cutoff scores for the TAS-20 are reported as follows: >61 indicates alexithymia, 51–61 indicates possible or borderline alexithymia, and <51 indicates no alexithymia. As a measure of alexithymia, the TAS-20 has strong convergent and concurrent validity, and moderate discriminant validity, in healthy adults.^51^ In adults with chronic pain, the TAS-20 is stable and valid; yet, the *Externally Oriented Thinking* subscale is not internally consistent; additionally, the 3-subscale structure has failed to be replicated in this population; thus, the questionnaire structure varies based on whether it is a healthy or clinical population.^52-55^

The AQC is another version of the TAS-20 that has specifically been modified for children and adolescents.^56^ The AQC is composed of the same three subscales as the TAS-20 and showed reliability and predictive ability for the first two subscales (*Difficulty Identifying Feelings, Difficulty Describing Feelings*) but low reliability for the third (*Externally Oriented Thinking*).^56^

The Emotional Awareness Questionnaire (EAQ) is a further adaptation of the AQC and consists of six subscales: *Differentiating Emotions, Verbal Sharing, Acting Out Emotions, Bodily Awareness*, (which assesses interoceptive sensibility; results discussed in the Interoception section), *Analyses of Emotions*, and *Attending to Other’s Emotions*.^21^ Higher scores indicate more emotional awareness and, therefore, less alexithymia. The EAQ subscales were developed using a principal axis factor analysis and showed good psychometric properties and predictive value.^21^ The EAQ was further adapted into the EAQ-revised (EAQ-R); the *Acting Out Emotions* subscale was dropped and another subscale, *Not Hiding Emotions*, was added.^22^ The AQC, EAQ, and EAQ-R, however, have not been validated in children and adolescents with chronic pain.

#### Results of individual studies.

##### Mean alexithymia in pain versus comparison group.

Ten studies reported mean alexithymia scores in a group of children with chronic pain, including persistent somatoform pain disorder, somatic symptom disorder, functional abdominal pain, and headaches. Eight of these also reported scores from a group without pain, but two had no comparison group.^37,50^

##### Chronic pain, persistent somatoform pain disorder, and somatic syndrome disorder.

Three studies reported mean alexithymia in participants with chronic pain, persistent somatoform disorder, or somatic symptom disorder (Table 2). Aaron et al. recruited participants with chronic pain (headache, musculoskeletal pain, stomach pain, etc.) from a children’s hospital and compared them with adolescents without pain (total *N* = 44).^39^ Adolescents with chronic pain had higher scores on the TAS-20 total score and *Difficulty Identifying Feelings* subscale. Heniquez et al. enrolled participants with somatic symptom disorder (*N* = 19, of which 17 were currently hospitalized).^37^ They reported results from the Children’s Toronto Alexithymia Scale. They did not enroll a comparison group.

Sayin et al. enrolled participants with stomachache or headache recruited from the pediatric neurology or gastroenterology departments in a university hospital. The study had two comparison groups: participants with depression recruited from the psychiatry department and a group of patients who had been hospitalized in the pediatric in-patient clinic who did not meet criteria for headache, abdominal pain, or any DSM-IV-TR psychiatric disorder (total *N* = 51).^40^ Authors used a yes/no version of the Toronto Alexithymia Scale, 26-item version (TAS-26). No significant differences were observed between groups on the TAS-26 total score; however, alexithymia scores were positively correlated with anxiety scores in the pain group. Only standardized *Z*-scores for the TAS-26 total score were reported with no raw values or subscale scores reported.

##### Functional abdominal pain.

Van der Veek et al. enrolled participants with functional abdominal pain who had been recruited from the pediatric gastroenterology clinic of a university hospital.^36^ The study also enrolled two comparison groups recruited from local schools: one group of children who had reported no abdominal pain in the past 2 weeks and another group of children who had reported some abdominal pain in the past 2 weeks (total *N* = 776). Mean scores on the three alexithymia subscales of the EAQ-R^22^ (*Differentiating Emotions, Verbal Sharing of Emotions*, and *Not Hiding Emotions*) were compared between the abdominal pain and the no-pain group. The abdominal pain group had lower emotional awareness (i.e., higher alexithymia) on all three of the subscales, when compared with the no-pain group.

##### Headache.

Six studies reported alexithymia scores in children with headaches. Gatta et al. recruited children with migraine headaches from a headache clinic and a comparison group without headaches recruited from routine medical visits (total *N* = 64).^46^ Alexithymia was assessed with the AQC. The mean scores of *Difficulty Identifying Feelings* and *Externally Oriented Thinking* and the total AQC score were significantly higher in the headache group, but the *Difficulty Identifying Feelings* score was not different.^46^ Cerutti et al. enrolled participants with migraine headaches and a comparison group recruited from schools (total *N* = 106). The total score and three subscale scores of the TAS-20 were significantly higher in the headache group compared with the non-headache group.^45^

Gatta et al. enrolled children with migraines or tension-type headaches recruited from a headache clinic and a comparison group of children without headaches recruited from routine medical visits (*N* = 121). Alexithymia assessed using the AQC was elevated in the tension-type headache group, when compared with both the migraine and control groups, as measured by total score and the *Difficulty Identifying Feelings* score. Scores on *Difficulty Identifying Feelings* and *Externally Oriented Thinking* were not different between groups.^47^

Natalucci et al. enrolled children with headaches and a comparison group of children with either anxiety or depression (*N* = 66).^49^ Alexithymia was assessed with the AQC. No differences were reported between the headache and comparison groups on any subscale or the total score. Natalucci et al. recruited children with migraines without aura from a headache clinic, as well as children without headaches (*N* = 161). They reported that the median *Difficulty Identifying Feelings* and *Difficulty Identifying Feelings* scores from the AQC were significantly higher in the headache group than the comparison group, but the *Externally Oriented Thinking* and total scores were not different.^48^ Gorobets et al. enrolled 84 adolescents with tension-type headaches or migraines.^50^ Mean score on the AQC was reported, but there was no comparison group.

#### Association between pain and alexithymia.

Four studies reported on the association between pain and alexithymia. Aaron et al. reported that pain bother-someness (or bother) and pain interference, but not pain intensity, were positively correlated with the *Difficulty Identifying Feelings* subscale of the TAS-20 in adolescents with chronic pain (Table 3).^39^ Gorobets et al. assessed alexithymia with the AQC and intensity of headaches with a visual analog scale. There was no significant correlation between total AQC score and headache intensity or duration.^50^ Hamdan-Mansour et al. measured alexithymia (TAS-20) and fibromyalgia, as determined by the London Fibromyalgia Epidemiology Study Screening Questionnaire (LFESSQ), in high school students (*N* = 483).^43^ The LFESSQ was used to diagnose participants with fibromyalgia; 9.9% of the participants met criteria. In this study, the total TAS-20 score did not predict the presence of fibromyalgia, when adjusting for psychological distress, age, and other demographic variables. Finally, among children with functional abdominal pain, Van der Veek et al. employed a structural regression model and found that the EAQ subscales *Analyses of Emotions* and *Attending to Other’s Emotions* were significantly associated with abdominal pain but the other subscales were not.^36^

#### Prevalence of alexithymia between groups.

Six studies reported on the differences between groups in those meeting the cutoff for alexithymia (Table 4, and details in Supplementary Appendix SA3). Five studies are described above, save the one by Wojtowicz et al., which enrolled *N* = 100 adolescents from a pain rehabilitation program with limb pain, headache, abdominal pain, and other forms of chronic pain.^41^ The majority of adolescents (59%) were judged to meet criteria for alexithymia, as assessed by clinicians. The process or questionnaire that was used to assess alexithymia was not described.

### Interoception

#### Study characteristics.

Two studies met criteria to be included in this review, both of which had alexithymia outcomes and were discussed in the Alexithymia section.^36,37^ Van der Veek et al. enrolled children with functional abdominal pain as well as children with no abdominal pain. The *Bodily* Awareness subscale of the EAQ-R was used to assess interoceptive sensibility, with higher scores indicating lower levels of interoception.^22^ The difference in interoceptive sensibility scores between the functional abdominal pain and no-pain group was significant, suggesting that children with functional abdominal pain had lower interoceptive sensibility compared with the no-pain group.

Heniquez et al. enrolled children with somatic symptom disorder (*N* = 19, of whom 17 were hospitalized) and assessed interoceptive sensibility with the Body Perception Questionnaire, assessed interoceptive accuracy with a heartbeat task, asked participants to rate their confidence on the heartbeat task on a 1–5 scale, and then calculated an interoceptive awareness score (the correspondence between their performance on the heartbeat task and their confidence in it).^37^ A comparison group without pain was not enrolled; thus, there were no statistical comparisons.

### Risk of bias

Nine of the 14 included studies were case–control designs and were assessed using an adaptation of the Newcastle–Ottawa scale (Supplementary Table SA4). On a six-point scale, with 6 representing lower risk of bias (and thus higher study quality), studies scored between 1 and 4.5 points. No study scored 6 points. The most common reason for being marked down was insufficient reporting of results; for example, failing to report confidence intervals for estimates or only reporting *p*-values as above/below a certain value rather than reporting an actual value. Gatta et al. and Natalucci et al. only discussed alexithymia questionnaire results narratively (i.e., significant/nonsignificant differences), without providing actual numerical results in a table, preventing use of those data for comparison or future meta-analysis. Sayin et al. reported only the total TAS-26 score, rather than the three individual subscales and total score, and the values were standardized, further preventing comparison with other studies or meta-analysis. Cohort studies^43^ and those without a comparison group were not evaluated.^37,41,44,50^

### Additional studies of interest

Five studies on interoceptive exposure interventions for pediatric chronic pain did not meet full inclusion criteria because they did not assess interoception directly but are detailed in Supplementary Appendix SA4.^57-61^ Briefly, interventions which involved inducing or imagining interoceptive sensations reduced pain intensity and pain distress in children and adolescents with chronic pain.

## Discussion

This is the first systematic review that investigated both alexithymia and interoception in children and adolescents with chronic pain. Overall, results reflect an emerging literature examining these constructs in children and adolescents representing clinical pain populations.

### Alexithymia

Fourteen studies reporting on alexithymia and chronic pain in children or adolescents with pain diagnoses such as fibromyalgia, headache, and other pain syndromes were identified. Overall, the studies reported: (1) higher mean alexithymia in chronic pain groups compared with non-pain groups (in six of eight studies) and (2) significantly higher incidence of alexithymia (using >61 as a cutoff on the TAS-20) in pain groups versus non-pain groups (in two of two studies). Our results in pediatric chronic pain populations align with the literature in adult pain populations; three reviews reported elevated mean alexithymia in adults with chronic pain compared with adults without,^9,10,38^ as well as a positive association between alexithymia and pain intensity and pain interference in adults with chronic pain.^38^ The latter finding aligns with our results of a positive association between alexithymia and pain interference; however, in our review, two studies found no association between alexithymia and pain intensity.^39,50^

It is difficult to parse out the psychological and biological contributors to pain intensity and interference. Differences in how individuals regard and respond to pain and differences in physiological pain processing can both influence sensitivity and response to pain. The associations between alexithymia and objective measures of pain have been assessed in adults with chronic pain, finding that higher alexithymia was associated with higher pain sensitivity,^62-64^ though this has not been examined in pediatric chronic pain populations.

One possible explanation can be related to the multidimensional nature of pain: the sensory and affective dimensions.^7,65^ The sensory dimension is associated with communication from physiological systems outside the central nervous system (CNS) to the CNS, through ascending pathways. This sensory dimension is thought to be processed in the somatosensory cortices and is referred to as pain intensity. Alternatively, the affective dimension is linked to the unpleasant experiences of pain, is thought to be processed in the insula and cingulate cortex, and reflects the threat or hedonic appraisal of sensation. The affective dimension of pain relies on the limbic system, which is essential in regulating and processing emotions. Results from cross-sectional and longitudinal brain imaging studies suggest that the chronification of back pain has been correlated with a shift away from acute pain circuits to the engagement of emotion circuits.^66,67^

Interestingly, a meta-analysis has reported that reduced insular and amygdalar volumes were associated with alexithymia, indicating shared neuro-networks between chronic pain and alexithymia.^68^ Furthermore, individuals with a high degree of alexithymia have limited emotional processing, as well as a greater difficulty in verbal communication of psychological distress, and hence, they may fail to enlist the aid or comfort of other people around them.^69^ This could lead to increased emotional distress, negatively affecting levels of depression and anxiety, resulting in greater pain. In addition, the dopamine system, which has roles in mood, emotion, and pain, has been investigated for its contribution. Gene polymorphisms in the enzyme that degradesdopamine (catechol-O-methyltransferase [COMT]) impact the association between alexithymia and pain sensitivity in healthy adults, though this has yet to be extended to chronic pain populations.^70^ Therefore, the mechanism by which alexithymia contributes to pain interference (or vice versa) in pediatric chronic pain requires further investigation. Though this review examined alexithymia in clinical pain populations, alexithymia is also associated with somatic symptoms in healthy samples of youth, and this association is partially mediated by depression.^71,72^ Future research in pediatric chronic pain should evaluate depression and its potential role in this association.

Though we identified 14 studies on alexithymia, results were not meta-analyzed for several reasons. First, incomplete reporting, as well as missing data, limited the data available for synthesis. Second, the heterogeneity in chronic pain diagnoses also limited the clinical utility of statistically synthesizing results (diagnoses included headache, fibromyalgia, abdominal pain, somatoform disorder, and more). Additionally, guidelines warn that meta-analyzing a small number of studies, or studies with small sample sizes (<100), can inflate effect estimates, yielding misleading results.^73,74^ Even if a meta-analysis had taken place, the variety of participant ages in the included studies may be problematic for estimating an effect size for an association, when there are likely developmental differences. The heterogeneity among the included studies limits the precision of our conclusions and precludes strong inferences about the strength or direction of associations in any single pain condition. However, the consistent patterns observed across diverse clinical populations suggest that the relationship between alexithymia and chronic pain may be broadly applicable. This diversity may enhance the generalizability of our findings, indicating that these constructs could be relevant across a range of chronic pain conditions rather than being limited to a specific diagnosis.

### Interoception

Only two studies were identified that reported measures of interoception in children with chronic pain. Both also included measures on alexithymia. In one study, interoceptive sensibility was significantly reduced in the pain group compared with the non-pain group. In the second study, the lack of a comparison group prevented statistical comparisons or meaningful conclusions. No other studies assessing interoception in children with chronic pain were identified.

Currently, clinical trials are generally expected or required to have prospectively written protocols and to publish results whether they align with or contradict study hypotheses. Recent recommendations for systematic review authors encourage the same level of transparency and completeness in reporting, supporting the publication of “empty reviews” with no studies or those with few studies. These reviews can identify important gaps in the literature, informing funding priorities, future trial design, and clinical decision-making by clarifying for clinicians when no evidence exists on an association or intervention.^30-32^ As such, though only two studies on interoception in children with chronic pain were identified, they were still included and assessed, as they identify an important gap in research. Future studies are warranted to further investigate the association between interoception and chronic pain in children and adolescents.

A recent theoretical review summarized the evidence for alterations in interoception in children with chronic pain and proposed a model in which altered interoception may predispose individuals to developing chronic pain through the development of maladaptive responses, brain changes, and autonomic dysregulation.^24^ For example, altered interoception is associated with lower heart rate variability (HRV) and changes in regions of the brain known to process interoceptive signals, while low HRV and these brain changes in turn correlate with chronic pain.^24^

Altered interoception may represent a transdiagnostic risk factor for psychiatric and neurological disorders.^15^ In depression and anxiety, symptoms may arise when changes in interoceptive states are not adequately anticipated, interpreted, and/or responded to.^15,75^ The literature has shown consistent alterations in interoception in individuals with anxiety,^75,76^ eating disorders,^77^ depression,^78^ autism spectrum disorder,^79,80^ and attention-deficit/hyperactivity disorder.^81^ However, studies varied in their assessment types (lab test, self-report questionnaire) and domains of interoception (accuracy, sensibility, etc.), thus comparison is difficult. Three reviews and/or meta-analyses on interoception in adults with chronic pain reported reduced interoceptive accuracy compared with adults without pain; thus, more research is needed on whether this association extends to pediatric chronic pain populations.^4-6^

#### Interventions to alter interoception.

Interventions have been developed specifically to increase adaptive interoceptive accuracy and awareness, including mindfulness approaches, breathing practices, vagus nerve stimulation, and more.^82^ One such intervention, Mindful Awareness in Body-Oriented Therapy (MABT), combines touch, mindfulness, and psychoeducation to cultivate interoceptive awareness.^83^ This intervention, delivered individually over 8 weeks, was shown to modulate neural networks involved in interoception in a pilot study in moderately stressed adults, and these increases in neural connectivity were associated with increased MAIA scores.^84^ These findings highlight brain plasticity in response to interoceptive training and suggest the need for future study of underlying mechanisms specific to interoceptive training. A recent review of interoception-based interventions in adults with chronic pain or mental health conditions (k = 31) found that 65% of included randomized controlled trials reported that these interventions were effective at improving interoception (mostly interoceptive sensibility), compared with control conditions.^85^ The most notable results were found in populations with fibromyalgia, among others. Another systematic review and meta-analysis on mindfulness-based interventions for chronic pain in adults found significant improvements in interoception (Becker’s *d* = 1.168, *p* < 0 .01) and reductions in pain intensity (*d* = −1.46, *p* = 0 .01) and pain interference (*d* = −1.07, *p* < 0.001) following the mindfulness-based interventions.^86^ Much more research is needed on interoception-based interventions in pediatric populations.

#### Quality of the evidence.

The included studies were of moderate quality; the main concern was incomplete reporting of statistical results. Several studies only narratively discussed results, without presenting data in tables. Others only presented point estimates without providing confidence intervals or standard error, which would allow assessment of the precision of the estimates. The incomplete reporting prevented detailed comparison between studies. Several studies enrolled a pain group without a comparison group without pain, limiting the comparisons that can be drawn.

#### Future directions: Developmental and theoretical considerations.

While the studies reviewed here show some evidence of differences between children and adolescents with and without chronic pain conditions, the majority of these studies have examined cross-sectional data in case–control studies. Additionally, most measures of interoception and alexithymia in youth have been adapted from measures developed in adult samples. As such, there has been minimal consideration of the natural course of the development of dimensions of interoception and emotional awareness across childhood. Somatosensory neurobiology evolves dramatically across development.^87,88^ It is possible that youth with chronic pain experience delays or disruptions in the development of the nervous system circuitry that underlies interoception. Interoceptive awareness also requires metacognition, which is known to develop well into late adolescence.^89-91^ Without a better understanding of the developmental norms for the neurocircuitry and cognitive abilities that are linked with interoception and how interoceptive processes emerge over time during childhood and adolescence, interpretation of study results in children and adolescents will remain somewhat limited.

**FIG. 2.**
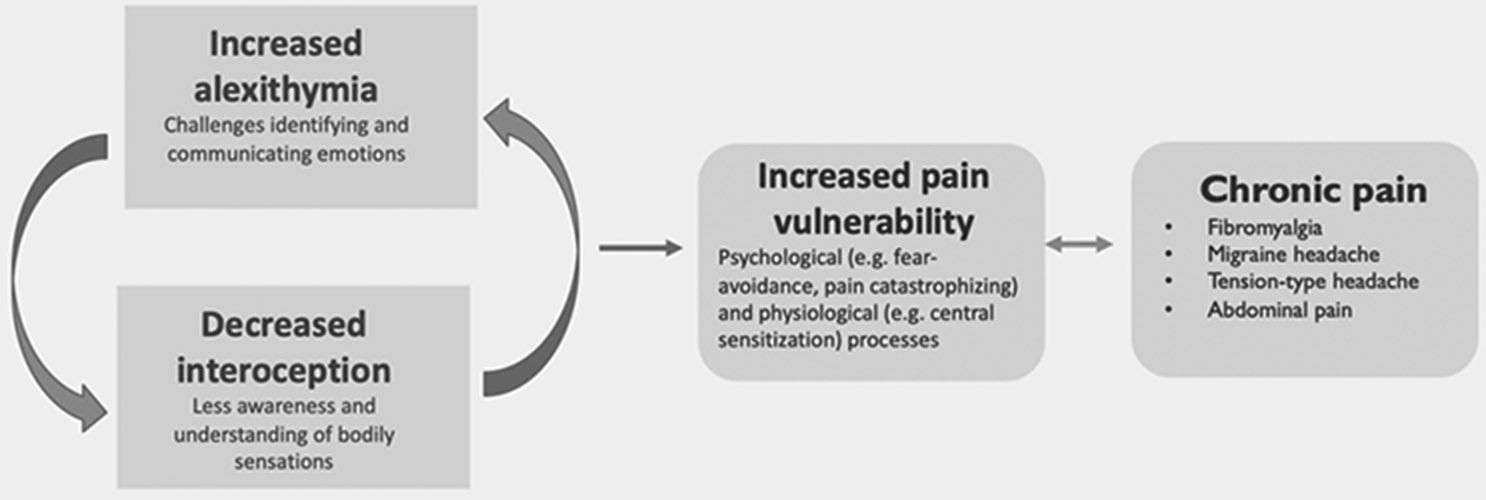
Possible role of alexithymia and interoception in chronic pain.

Additionally, while interoceptive awareness is generally conceptualized as a positive skill (i.e., more is better), overly high somatic symptoms or elevated awareness of/difficulty regulating awareness of bodily sensations are generally conceptualized as being problematic in the pediatric pain literature. This is reflected in research on somatic symptoms and anxiety sensitivity, which are thought to increase risk for the development of chronic pain.^92^ There are a number of related constructs that may also be important to explore in this area, including emotion regulation,^93^ anxiety sensitivity, and intolerance of uncertainty.^94^ One such construct is metacognition, the recognition of cognitive and emotional states (in oneself and in others) and the ability to influence and regulate them. Two of the studies included in this review found no difference in metacognition between pain and non-pain groups.^49,50^ Future work might also focus on integrating interoception and alexithymia into existing theoretical models of fear avoidance and interoceptive fear conditioning in pediatric pain, with consideration of potential shared mechanisms (Fig. 2).^92,95^

#### Strengths and limitations.

The strengths of this review include the comprehensive search strategy, the quality assessment, and prospectively written protocol. One limitation was the lack of a meta-analysis, which could be performed due to small number of studies and heterogeneity of the included studies. Another limitation of this review was the lack of a standardized definition of chronic pain. Chronic pain is usually defined as pain that persists, or recurs, over a period of 3 months or more.^1^ Whether duration of pain was an inclusion criterion for the participants of each included study was reported in Table 1. Six studies required that participants’ pain lasted for >3 or >6 months, but the remaining studies had different requirements or did not report if duration of pain was an inclusion criterion for the study.

Another limitation was that not all studies enrolled comparison groups of children without chronic pain. Some studies had no comparison groups at all. Several studies had comparison groups of other types; i.e., a pain group versus an anxiety/depression group or a chronic pain group versus a fibromyalgia group. This somewhat limits the generalizability and interpretability of results, since anxiety and depression have known associations to pain in pediatric populations.^96,97^

Limitations of the included studies were their cross-sectional nature, which limit the ability to determine causality and changes over time, as well as the lack of demographic information available on participants; only one study of the 14 reported the race and ethnicity of participants, limiting our ability to comment on the diversity of the samples.

## Conclusions

Alexithymia is elevated in children and adolescents with chronic pain. More research is needed on the role of altered interoception in pediatric chronic pain.

## Supplementary Material

Supplemental information

PRISMA checklist

Supplementary Data S1

Supplementary Data S2

## Figures and Tables

**Table 1:** Characteristics of included studies

Author, year	Country	Pain condition	N	Mean age	Gender	Diagnostic criteria	Duration of pain
Aaron 2019	US	chronic pain	PG: 22	16.0	74% F	NR	>3 months
			CG: 22	16.0	59% F		
Burba 2006	Lithuania	persistent somatoform pain disorder	PG: 120	NR	70% F	DSM-IV	NR
			CG: 60	NR	30% F		
Cerutti 2016	Italy	headaches (M)	PG: 53	13.4	57% F	ICHD-3-β	>6 months
			CG: 53	12.5	53% F		
Dell’Erba 2023§	Italy	fibromyalgia	PG: 21	13.6	43% F	ICHD-3/ ACR	>3 months
			CG: 16*	14.8	94% F		
Gatta 2011	Italy	headaches (TT)	PG: 32	11.2	81 % F	ICHD-2	>6 months
			CG: 32	11.8	81% F		
Gatta 2015	Italy	headaches (TT)	PG: 47	12.4	77% F	ICHD-3-β	>6 months
		headaches (M)	PG: 42	13.1	57% F		
			CG: 32	11.8	81% F		
Gorobets 2024	Russia	headaches	PG: 84	14	61% F	ICHD-3	NR
Heniquez 2022	France	somatic symptom disorder	PG: 19	13.8	74% F	DSM-5	NR
Hamdan-Mansour 2022	Jordan	fibromyalgia	483 total	16.2*	42% F	LFESSQ	>3 months
Natalucci 2019a¥	Italy	headaches (M)	PG: 34	11	41% F	ICHD-3-β / ICD-10	NR
			CG: 32	10	50 % F		
Natalucci 2019b	Italy	headaches (M)	PG: 70	10.2	49% F	ICHD-3-β	<3 years 51%; ≥3 years: 49%
			CG:70	10.6	56% F		
Sayin 2007	Turkey	headaches or abdominal pain	PG: 21	14.4	67% F	NR	NR
			CG: 15	12.4	NR		
Van der Veek 2012	Netherlands	functional abdominal pain	PG: 114	11.9	68% F	ROME-III	>8 weeks over the past 12 months
			CG: 255	14.4	33% F		
Wojtowicz 2014	US	chronic pain	PG: 100	15.8	79% F	NR	1-156 months

Note: ACR, American College of Rheumatology. CG, comparison group (PG, pain group). DSM-IV, Diagnostic and Statistical Manual of Mental Disorders, version 4. F, female gender. ICHD, International Classification of Headache Disorders. ICD-10, International Classification of Diseases-10th revision. LFESSQ, London Fibromyalgia Epidemiology Study Screening Questionnaire. M, migraine headache. NR, not reported. ROME-III, diagnostic criteria for irritable bowel syndrome. TT, tension-type headache. US, United States.

* Mean age reported for pain group and comparison group together.

§ Dell-Erba: a chronic pain group and comparison group with fibromyalgia.

¥ Natalucchi 2019: comparison group had either anxiety or depression.

**Table 2:** Mean (SD) alexithymia scores between pain and comparison groups

Study	Measure	Subscale	Pain group	Comparison group	P-value*		Conclusion
Aaron 2019	TAS-20	*DIF*	18.5 (5.3)	13.6 (3.8)	0.001	↑	Alexithymia was higher in pain group for 1 of 3 subscales and total score.
		*DDF*	14.7 (5.1)	12.6 (4.5)	0.14	--	
		*EOT*	19.6 (3.9)	19.0 (4.7)	0.68	--	
		*Total*	52.8 (12.2)	45.2 (11.4)	0.039	↑	
Cerutti 2016	TAS-20	*DIF*	16.4 (6.9)	12.8 (4.9)	<0.01	↑	Alexithymia was higher in pain group for all 3 subscales and total score.
		*DDF*	13.9 (3.7)	11.9 (3.4)	<0.05	↑	
		*EOT*	25.9 (5.5)	23.5 (5.7)	<0.01	↑	
		*Total*	56.3 (12.1)	48.2 (10.3)	<0.01	↑	
Gatta 2011	AQC	*DIF*	18.6 (5.6)	15.0 (5.3)	<0.05	↑	Alexithymia was higher in pain group for 2 of 3 subscales and total score
		*DDF*	15.3 (4.7)	13.9 (4.1)	ns	--	
		*EOT*	24.3 (4.2)	20.9 (4.2)	<0.05	↑	
		*Total*	58.1 (10.6)	49.8 (8.6)	<0.05	↑	
Gatta 2015¥	AQC	*DIF*	NR	NR	0.02	↑	Alexithymia was higher in pain group for 1 of 3 subscales and total score. Tension headache group had higher scores than migraine and no-pain group.
		*DDF*	NR	NR	ns	--	
		*EOT*	NR	NR	ns	--	
		*Total*	NR	NR	0.04	↑	
Gorobets 2024	AQC	Total	13 (8-19)**	--	--	--	No comparison group.
Heniquez 2023	C-TAS	DIF	7.6 (0.8)	--	--	--	No comparison group.
		DDF	4.6 (0.6)	--	--	--	
		EOT	7.6 (0.6)	--	--	--	
		Total	20.0 (1.4)	--	--	--	
Natalucchi 2019a†	AQC	*DIF*	NR	NR	ns	--	Alexithymia was not different between pain and anxiety/depression groups.
		*DDF*	NR	NR	ns	--	
		*EOT*	NR	NR	ns	--	
		*Total*	NR	NR	ns	--	
Natalucchi 2019b	AQC	*DIF*	7 (5,9)	6 (3,8)	0.045	↑	Alexithymia was higher in the pain group for 2 of 3 subscales but not total score.
		*DDF*	5 (4,7)	4 (2,6)	0.02	↑	
		*EOT*	7 (6,8)	8 (5,9)	0.52	--	
		*Total*	18.5 (16,23)	17 (14,22)	0.06	↑	
Sayin 2007‡	TAS-26	*Total*	−0.3	0.2	0.3	--	No difference between pain and no pain groups.
				−0.3			
Van der Veek 2012Ø	EAQ-R	*DE*	16.9 (3.1)	18.3 (2.3)	<0.001	↑	Alexithymia was higher in the pain group than the non-pain group on all 3 subscales.
		*VSH*	5.7 (1.8)	6.7 (1.5)	<0.001	↑	
		*NHE*	9.6 (2.6)	10.6 (2.3)	0.001	↑	

* P-value for the difference between groups.

** Gorobets reported mean(range).

† Natalucchi 2019a: comparison group had either anxiety or depression.

‡ Sayin: study had a pain group, comparison group without pain, and a depression group.

Ø Van der Veek had a pain group, a some-pain group, and a no-pain group.

AQC, Alexithymia Questionnaire for Children; high score indicates more alexithymia. DDF, Difficulty describing feelings subscale. DE, Differentiating emotions subscale. DIF, Difficulty identifying feelings subscale. EAQ, Emotional Awareness Questionnaire; EAQ-R, Emotional Awareness Questionnaire-revised; lower score indicates more alexithymia. EOT, Externally-oriented thinking subscale. NHE, not hiding emotions subscale. Ns, not significant (p>0.05; exact p-value not reported). SD, standard deviation. TAS-20, Toronto Alexithymia Scale, 20-item version; higher score indicates more alexithymia. TAS-26, Toronto Alexithymia Scale, 26-item version; higher score indicates more alexithymia. VSE, Verbal sharing of emotions subscale. C-TAS, Children’s Toronto Alexithymia Scale.

↑ Alexithymia higher in pain group.↓ Alexithymia lower in pain group. -- Alexithymia not different between groups

**Table 3:** Association between alexithymia and pain

Study	Alexithymia measure	Pain measure	Results
Aaron 2019	TAS-20	Pain bothersomeness	The DIF subscales of the TAS was positively correlated with pain bothersomeness (r=0.55, p=0.02) and pain interference (r=0.55; p=0.02) but not pain intensity. The other subscales and total score were not significantly correlated.
		Pain interference	
		Average pain intensity	
Gorobets 2024	AQC	Pain intensity, pain duration	Total AQC score not correlated with headache intensity or duration.
Hamdan-Mansour 2022	TAS-20	LFESSQ	In a logistic regression, total TAS-20 score did not predict the existence of fibromyalgia, when adjusting for psychological distress and demographic characteristics.
Van der Veek 2012	EAQ	API	In a structural regression model, *Analyses of Emotions* and *Attending to Other’s Emotions* were significantly associated with abdominal pain, but the other subscales were not.

API, Abdominal Pain Index. EAQ, Emotional Awareness Questionnaire. LFESSQ, London Fibromyalgia Epidemiology Study Screening Questionnaire. SCL, Somatic Complaints List. TAS-20, Toronto Alexithymia Scale, 20-item.

**Table 4:** Percentage of participants meeting criteria for alexithymia, by group

Study	Group	N	Alexithymia	Borderline	No alexithymia	Test for differences between groups
Burba 2006*	PSPD	120	59%	--	41%	Fisher’s exact test: p<0.001
	No pain	60	1%	--	99%	
Cerutti 2016	Headache (M)	53	32%	34%	34%	*X*2-test: *X*2(2)=7.97; p=0.05.
	No pain	53	15%	25%	60%	
Dell’Erba 2022	Chronic pain	21	57%	0%	43%	--
	Fibromyalgia	16	56%	13%	31%	
Gatta 2011	Headache (TT)	32	44%	34%	33%	--
	No pain	32	9%	41%	50%	
Gatta 2015	Headache (M)	42	12%	24%	64%	--
	Headache (TT)	47	38%	24%	38%	
	No pain	32	9%	41%	50%	
Wojtowicz 2015	Chronic pain	100	59%	--	41%	--

M, migraine headache. PSPD, persistent somatoform pain disorder. TT, tension-type headache. *Burba 2006 only reported two categories: alexithymia and no alexithymia.

## Data Availability

Only publicly available data from previously published research were used for this study.

## Abbreviations Used

AQC: Alexithymia Questionnaire for Children
CINAHL: Cumulative Index to Nursing and Allied Health
COMT: catechol-O-methyltransferase
DSM-IV-TR: Diagnostic & Statistical Manual of Mental Disorders, 4th edition, Text Revision
EAQ: Emotional Awareness Questionnaire
EAQ-R: Emotional Awareness Questionnaire Revised
HRV: heart rate variability
LFESSQ: London Fibromyalgia Epidemiology Study Screening Questionnaire
MABT: Mindful Awareness in Body-Oriented Therapy
MAIA: Multidimensional Assessment of Interoceptive Awareness
PRISMA: Preferred Reporting Items for Systematic Reviews & Meta-Analyses
PROSPERO: Prospective Register of Systematic Reviews
TAS-20: Toronto Alexithymia Scale, 20-item version
TAS-26: Toronto Alexithymia Scale, 26-item version
