# Supplementary material for: The Association Between Alexithymia, Interoception, and Pain in Pediatric Chronic Pain: A Systematic Review
Source: Integr Med Rep. Author manuscript; Available in PMC 2025 Dec 31. (PMC12551025; doi:10.1177/27683222251375956)
Supplement: Supplemental information [file NIHMS2114112-supplement-Supplemental_information.docx]

**SUPPLEMENTARY INFORMATION**

**Appendix 1**: *Data extracted from the included studies*

**Table S1**: *Protocol deviations*

**Appendix 2**: *Examples of search strategy*

**Table S2**: *Adaptation of the Newcastle-Ottawa scale for assessing study quality*

**Table S3**: *Studies excluded after reading full text*

**Table S4:** *Newcastle-Ottawa scale for assessing the quality of the included studies*

**Appendix 3**: *Percentage of participants meeting criteria for alexithymia, further information*

**Appendix 4**: *Additional studies of interest which did not meet inclusion criteria*

**Appendix 1**: *Data extracted from the included studies*

Data extracted from each individual study included the following: first author and year; country in which study was conducted; chronic pain sample details (mean age and age range, male to female ratio, and race and ethnicity); diagnostic status of participants (type of pain diagnosis, duration of pain); co-occurring diagnoses of participants i.e., anxiety, depression, history of trauma, PTSD, ASD, ADHD, or other; comparison group (if present; mean age and age range, diagnostic status, i.e., screened to be pain-free); outcome measures (self-report, collateral report, or behavioral); medication status (*N* currently medicated, type of medication); if an intervention study, intervention dose and duration, adverse events or negative effects of intervention, and changes in pain, alexithymia, or interoception following intervention.

| **Table S1:** Protocol deviations, with rationale | | |
| --- | --- | --- |
| **#** | **Deviation from protocol** | **Rationale** |
| 1 | Did not clarify whether review would proceed if we identified no eligible studies. We decided we would. | Empty reviews can identify important gaps in the literature, inform funding priorities and provide clinically useful information. |
| 2 | Included alexithymia-- originally we only had interoception | Although we wanted to continue with the empty review (no interoception studies) we realize that journals generally don't like to publish empty reviews. We decided to expand the focus of our review to include alexithymia. |
| 3 | The original protocol stated that included studies were required to have measures of pain, but ultimately only a few studies did this, so we included studies which met the other criteria regarding outcome measures, namely, that they included measures of alexithymia or interoception in pain conditions. Pain data, when reported, was included and discussed in the review. |  |
| 4 | Though our protocol said English language studies only, we were able to translate the study using free translation software through the Chrome browser. We clarified that we did not exclude grey literature from the review if it was otherwise eligible. | Protocol said we would not include studies in languages other than English due to lack of resources for translation, but free translation software is now available. Original protocol did not mention whether grey literature was included or excluded. |

**Appendix 2**: *Examples of search strategy*

CINAHL

(TX interoception OR TX interoceptive OR TX interoceptor OR TX body awareness OR TX heartbeat tracking OR heartbeat perception) AND (TX pain OR TX chronic pain OR TX low back pain OR TX headache OR TX fibromyalgia OR TX somatoform OR TX TMJ OR TX abdominal pain OR TX tempomandibular OR TX migraine* OR TX functional pain OR TX neuropathic pain)

(alexithym*) AND (TX pain OR TX chronic pain OR TX low back pain OR TX headache OR TX fibromyalgia OR TX somatoform OR TX TMJ OR TX abdominal pain OR TX tempomandibular OR TX migraine* OR TX functional pain OR TX neuropathic pain)

PubMed/MEDLINE

(("Interoception"[Mesh] OR interoception[Title/Abstract] OR interoceptor*[Title/Abstract] OR interocept*[Title/Abstract] OR body awareness[Title/Abstract] OR heartbeat tracking[Title/Abstract] OR heartbeat perception[Title/Abstract])) AND ((pain[Title/Abstract] OR musculoskeletal[Title/Abstract] OR low back pain[Title/Abstract] OR headache[Title/Abstract] OR fibromyalgia[Title/Abstract] OR somatoform[Title/Abstract] OR TMJ[Title/Abstract] OR TMJD[Title/Abstract] OR tempomandibular[Title/Abstract] OR headache[Title/Abstract] OR migraine[Title/Abstract] OR migraineur[Title/Abstract] OR neuropathic pain[Title/Abstract] OR functional pain[Title/Abstract] OR CRPS[Title/Abstract] OR abdominal pain[Title/Abstract]))

*Filter applied: Child: birth-18 years.*

(("alexithymia[Title/Abstract] OR alexithy*[Title/Abstract])) AND ((pain[Title/Abstract] OR musculoskeletal[Title/Abstract] OR low back pain[Title/Abstract] OR headache[Title/Abstract] OR fibromyalgia[Title/Abstract] OR somatoform[Title/Abstract] OR TMJ[Title/Abstract] OR TMJD[Title/Abstract] OR tempomandibular[Title/Abstract] OR headache[Title/Abstract] OR migraine[Title/Abstract] OR migraineur[Title/Abstract] OR neuropathic pain[Title/Abstract] OR functional pain[Title/Abstract] OR CRPS[Title/Abstract] OR abdominal pain[Title/Abstract]))

*Filter applied: Child: birth-18 years.*

Scopus & PsychINFO

(interoception OR interoceptive OR interoceptor OR body AND awareness OR heartbeat AND tracking OR heartbeat AND perception) AND (pain OR musculoskeletal OR low back pain OR headache OR fibromyalgia OR somatoform OR TMJ OR TMJD OR tempomandibular OR migraine OR migraineur OR neuropathic pain OR functional pain OR CRPS OR abdominal pain)

(alexithym*) AND (pain OR musculoskeletal OR low back pain OR headache OR fibromyalgia OR somatoform OR TMJ OR TMJD OR tempomandibular OR migraine OR migraineur OR neuropathic pain OR functional pain OR CRPS OR abdominal pain)

**Table S2**: *Adaptation of the Newcastle-Ottawa scale for assessing study quality*

| **Item 1**: Adequacy of pain or diagnostic characterization  Were participants in the pain group diagnosed with a pain condition based on a standardized assessment, like the DSM-5 or ICHD?  *1 point with standardized assessment (DSM, ICHD, etc.)*  *0 points for no standardized assessment*  *(Note: Diagnosis can be made previously or reconfirmed for the study.)* |
| --- |
| **Item 2:** Selection of comparison group  Were comparison group participants matched on gender and age or other key factors, or were gender/age not significantly different between groups?  *1 point if an attempt was made to recruit groups similar in terms of gender and age (i.e., “recruited age and gender-matched comparison group.”)*  *0.5 points if they examined and reported the statistical significance of differences in age and gender between groups (most likely in Table 1) whether or not there were differences.*  *0 points if they did neither of these things.* |
| **Item 3**: *Definition of comparison group*  Clear pain-related inclusion and exclusion criteria for comparison group.  *1 point if controls have no history of pain previously diagnosed (ex: the participants section mentions that a questionnaire/interview asked about pain conditions) OR the exclusion criteria mention pain*  *0 points if no description (not sure that pain conditions has been formally excluded in the control group)* |
| **Item 4**: *Use of validated measures*  Were outcome measures on alexithymia and interoception validated for use in children and adolescents?  *1 point if outcome is a validated tool or questionnaire or objective test of interoception or alexithymia*  *0 points if outcome is not objective test or not a questionnaire validated for this population (children/adolescents)* |
| **Item 5**: *Sample size and power*  Were sample size and power calculations performed *a priori?*?  *1 point if a priori sample size/power calculation performed and reported.*  *0 points if no sample size/power calculation.* |
| **Item 6:** *Statistical tests and reporting*  Were statistical tests clearly described, appropriate, and data presented in full (i.e., including measure of association or effect, confidence interval, and p-value)?  *1 point if the statistical test(s) used to analyze the data is/are clearly described, appropriate, and presented in full (i.e., including measure of association, confidence interval, and p-value).*  *0 points if statistical test is not appropriate, not described, or incomplete.* |

| **Table S3**: Studies excluded after reading full text | | |
| --- | --- | --- |
| Study | Rationale |  |
| Alslman E, Thultheen I, Hamaideh SH, Nofal B, Hamdan-Mansour R, Hamdan Mansour A. The mediating effect of psychological distress and bullying victimization on the relationship between alexithymia and fibromyalgia among school adolescents. MHSI. 2024;28(5):842-856. | No key outcomes- secondary data analysis from another study which was included in the review (Hamdan-Mansour) with no new relevant data. Authors confirmed this. |  |
| Camerota F, Mariani R, Petronelli G, et al. Affect regulation capabilities in hypermobility ehlers danlos syndrome: exploring the associations with pain perception and psychophysical health. Brain Sciences. 2025;15(2):202. | Wrong population (no chronic pain) |  |
| Cerutti, R., Spensieri, V., Valastro, C., Presaghi, F., Canitano, R., & Guidetti, V. (2017). A comprehensive approach to understand somatic symptoms and their impact on emotional and psychosocial functioning in children. PloS one, 12(2), e0171867. | Wrong population (no chronic pain) |  |
| Fazia G, Carbone EA, Rania M, et al. Pain experience in eating disorders: The mediating role of depression, alexithymia and interoceptive awareness. Euro Eating Disorders Rev. 2024;32(1):148-160. | Wrong outcome (no alexithymia or interoception measure) |  |
| Grimsgaard E, Eik H, Bjorbækmo W, Ahlsen B. A breathing space: how young Norwegian women engaging psychomotor physiotherapy to address long-term health disorders narrate their experiences. Physiotherapy Theory and Practice. 2025;41(4):772-782. | Wrong outcome (no alexithymia or interoception measure) |  |
| Hocoglu ZI, Aslan AT, Asfuroglu P, Cakir EP, Acar ASS, Eyuboglu TS. P359 “No words for feelings” The factors effecting alexithymia in the patients with cystic fibrosis and their mothers. Journal of Cystic Fibrosis. 2023;22:S174. | Wrong population (no chronic pain) |  |
| Ives, Lindsay Taylor (2023). Understanding Psychosocial Treatment Non-Response in Children with Functional Abdominal Pain. Dissertation, Duke University. Retrieved from  https://hdl.handle.net/10161/29190. | Wrong population (no chronic pain) |  |
| Jellesma FC, Rieffe C, Terwogt MM, Westenberg PM. Children's sense of coherence and trait emotional intelligence: a longitudinal study exploring the development of somatic complaints. Psychol Health. 2011;26(3):307-320. doi:10.1080/08870440903411021 | Wrong population (no chronic pain) |  |
| Jellesma, F. C., Rieffe, C., Terwogt, M. M., & Westenberg, M. (2009). Do I feel sadness, fear or both? Comparing self-reported alexithymia and emotional task-performance in children with many or few somatic complaints. Psychology & Health, 24(8), 881–893. | Wrong population (no chronic pain) |  |
| Lanzara R, Conti C, Lalli V, et al. Emotions in search of words: Does alexithymia predict treatment outcome in chronic musculoskeletal pain? Stress and Health. 2024;40(5):e3436. | Wrong population (adults). |  |
| Marzilli, E. (2017). Somatoform Symptoms In Adolescents Who Visit The Emergency Department: Alexithymia And Defensive Strategies. In Z. Bekirogullari, M. Y. Minas, & R. X. Thambusamy (Eds.), Health and Health Psychology - icH&Hpsy 2017, vol 30. European Proceedings of Social and Behavioural Sciences (pp. 257-266). Future Academy. https://doi.org/10.15405/epsbs.2017.09.24 | Wrong population (15-24 years old, data for adolescents and adults not presented separately) |  |
| Nicoli MS, Tarantino G, Marasco E, et al. Pos0282 patients with juvenile idiopathic arthritis (Jia) on intensive treatment frequently present alexithymia over fatigue, anxiety, and depression, despite low disease assessment parameters. In: *Scientific Abstracts*. BMJ Publishing Group Ltd and European League Against Rheumatism; 2023:382.1-382. | Poster presentation; paper not published yet. |  |
| Opdensteinen KD, Rach H, Gruszka P, et al. “The mere imagination scares me”—evidence for fear responses during mental imagery of pain-associated interoceptive sensations in adolescents with chronic pain. Pain. 2024;165(3):621-634. | Wrong outcome (no alexithymia or interoception measure) |  |
| Rieffe C, De Rooij M. The longitudinal relationship between emotion awareness and internalising symptoms during late childhood. Eur Child Adolesc Psychiatry. 2012;21(6):349-356. doi:10.1007/s00787-012-0267-8 | Wrong population (no chronic pain) |  |
| Rieffe C, Terwogt MM, Bosch JD, Frank Kneepkens CM, Douwes AC, Jellesma FC. Interaction between emotions and somatic complaints in children who did or did not seek medical care. Cognition & Emotion. 2007;21(8):1630-1646. | Wrong outcome (no alexithymia or interoception measure) |  |
| Rieffe, C., Oosterveld, P., Meerum Terwogt, M., Novin, S., Nasiri, H., & Latifian, M. (2010)Relationship between alexithymia, mood and internalizing symptoms in children and young adolescents: Evidence from an Iranian sample. Personality and Individual Differences, 48, 425-430. | Wrong population (no chronic pain) |  |
| Rieffe, C., Terwogt, M. M., & Bosch, J. D. (2004). Emotion understanding in children with frequent somatic complaints. European Journal of Developmental Psychology, 1(1), 31e47. | Wrong outcome (no alexithymia or interoception measure) |  |
| Rieffe, C., Villanueva, L., Adrián, J.E., & Górriz, A.B. (2009). Quejas somáticas, estados de ánimo y conciencia emocional en adolescentes [Somatic complaints, mood states and emotional awareness in adolescents]. Psicothema, 21, 459-464. | Wrong population (no chronic pain) |  |
| Skovslund Nielsen E, Kallesøe K, Bennedsen Gehrt T, et al. Trajectories of change, illness understanding, and parental worries in children and adolescents undergoing internet-delivered cognitive-behavioral therapy for functional abdominal pain disorders: protocol for a single-case design and explorative pilot study. JMIR Res Protoc. 2025;14:e58563. | Protocol for a study; author confirmed study was not published and data were not available. |  |
| Tozzi E, Andrisani G, Maiorani D, Pezzi L, Leopardi R, Fiorentini N, Sechi E. Alexithymia in headache sufferers: A psychosomatic symptom or comorbidity in adolescents? Educational Research. 2013; 4(3), 286-293. | Wrong population (included children and adults). |  |
| van de Putte EM, Engelbert RH, Kuis W, Kimpen JL, Uiterwaal CS. Alexithymia in adolescents with chronic fatigue syndrome. *J Psychosom Res*. 2007;63(4):377-380. doi:10.1016/j.jpsychores.2007.07.009 | Wrong population (no chronic pain) |  |
| Van der Veek, S. M. C., Derkx, H. H. F., De Haan, E., Benninga, M. A., & Boer, F. (2010). Abdominal pain in Dutch school children: relations with physical and psychological comorbid complaints in children and their parents. Journal of Pediatric Gastroenterology and Nutrition, 51(4), 481e487. | Wrong outcome (no alexithymia or interoception measure) |  |

| **Table S4:** *Newcastle-Ottawa scale for assessing the quality of the included studies* | | | | | |  |  |
| --- | --- | --- | --- | --- | --- | --- | --- |
|  | **Adequacy of pain diagnosis** | **Selection of comparison group** | **Definition of comparison group** | **Use of validated measures** | **Sample size and power** | **Statistical reporting** | **Total score** (of 6) |
| Aaron 2019 | 0 | 0.5 | 1 | 1 | 1 | 1 | **4.5** |
| Burba 2006 | 1 | 0 | 0 | 1 | 0 | 1 | **3** |
| Cerutti 2016 | 1 | 0 | 1 | 1 | 0 | 0 | **3** |
| Gatta 2011 | 1 | 1 | 1 | 1 | 0 | 0 | **4** |
| Gatta 2015 | 1 | 0 | 1 | 1 | 0 | 1 | **4** |
| Natalucci 2019a | 1 | 0 | 0 | 1 | 0 | 0 | **2** |
| Natalucci 2019b | 1 | 1 | 1 | 1 | 0 | 1 | **5** |
| Sayin 2007 | 0 | 0 | 0 | 1 | 0 | 0 | **1** |
| Van der Veek 2012 | 1 | 0.5 | 1 | 1 | 0 | 1 | **4.5** |
|  | | | | | | | |

**Appendix 3**: *Percentage of participants meeting criteria for alexithymia, further information*

*Chronic pain*

In a study in adolescents 12-17 with persistent somatoform pain disorder, 59% of the pain group met criteria for alexithymia while 41% did not.^1^ In the comparison group of healthy adolescents without pain, 1% met criteria for alexithymia and 99% did not. This difference in the prevalence of alexithymia between groups was statistically significant (Fisher’s exact test; p<0.001). In a descriptive study in 100 pediatric participants (age range not reported; mean age = 15.8) with chronic pain defined as limb pain, headache, abdominal pain, or other pain, 59% had alexithymia based on clinician evaluation.^2^ A comparison group was not enrolled in this study.

*Fibromyalgia*

A study in participants 13-18 with chronic pain (n=21) and juvenile fibromyalgia (n=16) reported a similar prevalence of alexithymia between groups.^3^ In the chronic pain group, 57% of participants reported symptoms of alexithymia, 0% were borderline, and 43% reported no symptoms of alexithymia. In the juvenile fibromyalgia group, 56% reported symptoms of alexithymia, 13% borderline, and 31% reported no symptoms of alexithymia.

*Headache*

There were three studies which enrolled participants with headaches and reported the proportion of participants who met criteria for alexithymia and borderline alexithymia. In the first headache study, for the migraine headache group, 32% of participants met criteria for alexithymia, 34% for borderline alexithymia, and 34% did not meet criteria for either.^4^ In the comparison group without headaches, the percentages were 15%, 24.5%, and 60.5%, respectively. This difference between groups was statistically significant (*X*2_(2)_=7.97; p=0.05). None of the other headache studies examined the between-group difference statistically.

In the second headache study, in the tension-type headache group, 44% of participants met criteria for alexithymia, 34% for borderline alexithymia, and 22% for neither. In the comparison group without headaches, the percentages were 9%, 41%, and 50%, respectively.^5^ The third headache study enrolled participants with migraine headaches or tension-type headaches, and a comparison group with no headaches. In the migraine group, 12% of participants met criteria for alexithymia, 24% for borderline alexithymia, and 64% for neither. In the tension-type headache group, they were 38%, 24%, and 38%; in the comparison group they were 9%, 41% and 50%.^6^

**Appendix 4**: *Additional studies of interest which did not meet inclusion criteria*

Zucker et al. (2017) conducted a single-arm trial to evaluate effects of a 10-week acceptance-based interoceptive exposure of parent-child dyads (*n*= 24 dyads). Using cartoon characters to represent bodily sensations (e.g., Gassy Gus), children were trained to be ‘FBI agents’ (Feeling and Body Investigators) to facilitate a curious stance and focus on somatic symptoms.^7^ Parent and child ratings of child pain, the primary outcome, were obtained three times daily for a 14-day pretreatment period and, equivalently, for a 14-day post treatment period. Patient-reported pain (experience, distress, and interference) and negative affect had clinically and statistically significant reductions with effect sizes ranging from 0.48–71 for pain and from 0.38–.61 for pain distress.

Two clinical case studies have been published. Dobe et al. (2009) described and evaluated effects of Pain Provocation Technique (PPT) with a 15-year-old with chronic pain and severe emotional distress receiving care at a pediatric palliative facility.^8^ PPT was described as a pain-management strategy for children suffering from chronic pain, pain related fear, and feelings of helplessness toward their pain, and incorporates the following components: (a) interoceptive exposure, (b) bilateral stimulation in the form of tapping, and (c) cognitive coping strategies and imagery techniques to reduce the pain intensity. Authors suggested that effects of a treatment indicated a decrease in pain intensity and emotional distress a 3-, 6, and 12-month follow-up. Finally, Allen et al. (2012) published two clinical case reports describing the use of a transdiagnostic protocol for treatment of two adolescents with chronic pain (headaches and wide-spread pain) and mood disorders. The protocol was described as a flexible, modular-based individual treatment protocol with 8 to 20, 50-minute sessions that employed the application of general cognitive-behavioral strategies to pain and anxiety and instead focuses on identifying and modifying maladaptive emotion regulation and response strategies associated with the experience of pain. Clinically significant improvement in emotional symptoms, emotion regulation skills, somatization, and functional disability were reported.^9^

A trial investigating the PPT as adjunctive treatment to multimodal therapy (education, coping strategies, physical exercise) in N=80 adolescents with chronic pain, comparing it to multimodal therapy alone.^10^ The multimodal + PTT group had a significant decrease in pain intensity over the 3-month intervention compared to the multimodal group alone (F=6.52; p=0.013). Flack et al. (2017) used a sample of adolescents (age 11-18) with either chronic headaches (N= 20) or chronic abdominal pain (N= 20) to examine how interoceptive sensations located distally or locally to the adolescent’s pain region was associated with self-reported fear and avoidance.^11^ Adolescents were trained to tighten the muscles in three areas of their body for up to 3 minutes each to induce interoceptive sensations: (1) stomach (distal to headache pain, proximal to abdominal pain); (2) frowning (distal to abdominal pain, proximal to headache pain); and (3) fists (safe comparison task). Proximal interoceptive sensations were associated with higher self-reported fear and avoidance for adolescents in the chronic abdominal pain group compared to the distal and safe comparison tasks. Proximal interoceptive sensations were associated with higher self-reported avoidance for adolescents in the chronic headache group compared to the safe comparison task.

Finally, Flack et al. (2018) used a sample of 127 adolescents (age 11-17) with a range of chronic pain diagnoses who were receiving three weeks of intensive interdisciplinary pain treatment (IIPT).^12^ Half of the participants were randomized to receive interoceptive exposure in addition to IIPT, which consisted of adolescents provoking an increase in pain intensity by focusing on their pain and pain-related memories, and then using pain coping strategies to reduce their pain intensity. The other half were randomized to receive relaxation therapy, consisting of a modified progressive muscle relaxation protocol, in addition to IIPT. All participants, regardless of treatment group, showed a decrease in fear of pain, pain intensity, pain-related disability, anxiety sensitivity, and pain catastrophizing both at the end of the 3-week treatment and at a 3-month follow-up. However, interoceptive exposure was related to greater decreases in fear of pain than the relaxation therapy group for participants with higher fear of pain prior to treatment and for participants with abdominal pain.

**REFERENCES**

1. Burba B, Oswald R, Grigaliunien V, et al. A controlled study of alexithymia in adolescent patients with persistent somatoform pain disorder. Can J Psychiatry Rev Can Psychiatr 2006;51(7):468–471; doi: 10.1177/070674370605100709.

2. Wojtowicz AA, Banez GA. Adolescents with chronic pain and associated functional disability: A descriptive analysis. J Child Health Care Prof Work Child Hosp Community 2015;19(4):478–484; doi: 10.1177/1367493514523157.

3. Dell’Erba S, Melissano P, Zegretti A, et al. Psychological characteristics of juvenile fibromyalgia syndrome. Pediatr Int Off J Jpn Pediatr Soc 2023;65(1):e15449; doi: 10.1111/ped.15449.

4. Cerutti R, Valastro C, Tarantino S, et al. Alexithymia and psychopathological symptoms in adolescent outpatients and mothers suffering from migraines: a case control study. J Headache Pain 2016;17:39; doi: 10.1186/s10194-016-0640-y.

5. Gatta M, Canetta E, Zordan M, et al. Alexithymia in juvenile primary headache sufferers: A pilot study. J Headache Pain 2011;12(1):71–80; doi: 10.1007/s10194-010-0248-6.

6. Gatta M, Spitaleri C, Balottin U, et al. Alexithymic characteristics in pediatric patients with primary headache: a comparison between migraine and tension-type headache. J Headache Pain 2015;16:98; doi: 10.1186/s10194-015-0572-y.

7. Zucker N, Mauro C, Craske M, et al. Acceptance-based interoceptive exposure for young children with functional abdominal pain. Behav Res Ther 2017;97:200–212; doi: 10.1016/j.brat.2017.07.009.

8. Dobe M, Hechler T, Zernikow B. The Pain Provocation Technique as an Adjunctive Treatment Module for Children and Adolescents with Chronic Disabling Pain: A Case Report. J Child Adolesc Trauma 2009;2(4):297–307; doi: 10.1080/19361520903317287.

9. Allen LB, Tsao JCI, Seidman LC, et al. A Unified, Transdiagnostic Treatment for Adolescents With Chronic Pain and Comorbid Anxiety and Depression. Cogn Behav Pract 2012;19(1):56–67; doi: 10.1016/j.cbpra.2011.04.007.

10. Hechler T, Dobe M, Damschen U, et al. The pain provocation technique for adolescents with chronic pain: preliminary evidence for its effectiveness. Pain Med Malden Mass 2010;11(6):897–910; doi: 10.1111/j.1526-4637.2010.00839.x.

11. Flack F, Pané-Farré CA, Zernikow B, et al. Do Interoceptive Sensations Provoke Fearful Responses in Adolescents With Chronic Headache or Chronic Abdominal Pain? A Preliminary Experimental Study. J Pediatr Psychol 2017;42(6):667–678; doi: 10.1093/jpepsy/jsw108.

12. Flack F, Stahlschmidt L, Dobe M, et al. Efficacy of adding interoceptive exposure to intensive interdisciplinary treatment for adolescents with chronic pain: a randomized controlled trial. Pain 2018;159(11):2223–2233; doi: 10.1097/j.pain.0000000000001321.
